# The impact of patient safety culture and the leader coaching behaviour of nurses on the intention to report errors: a cross-sectional survey

**DOI:** 10.1186/s12912-020-00472-4

**Published:** 2020-09-21

**Authors:** Zahra Chegini, Edris Kakemam, Mohammad Asghari Jafarabadi, Ali Janati

**Affiliations:** 1 Social Determinants of Health Research Center, Qazvin University of Medical Sciences, Shahid Bahonar Blvd, Zip code, Qazvin, 1531534199 Iran; 2 National Institute for Health Research, Tehran University of Medical Sciences, Tehran, Iran; 3 Department of Health Management and Economics, School of Public Health, Tehran University of Medical Sciences, Tehran, Iran; 4 Road Traffic Injury Research Center, Tabriz University of Medical Sciences, Tabriz, Iran; 5 Department of Statistics and Epidemiology, Faculty of Health, Tabriz University of Medical Sciences, Tabriz, Iran; 6 Tabriz Health Services Management Research Center, Tabriz University of Medical Sciences, Tabriz, Iran

**Keywords:** Coaching, Patient safety culture, Medical error, Cross-sectional studies, Iran

## Abstract

**Background:**

There is growing interest in examining the factors affecting the reporting of errors by nurses. However, little research has been conducted into the effects of perceived patient safety culture and leader coaching of nurses on the intention to report errors.

**Methods:**

This cross-sectional study was conducted amongst 256 nurses in the emergency departments of 18 public and private hospitals in Tabriz, northwest Iran. Participants completed the Hospital Survey on Patient Safety Culture (HSOPSC), Coaching Behavior Scale and Intention to Report Errors’ questionnaires and the data was analyzed using multiple linear regression analysis.

**Results:**

Overall, 43% of nurses had an intention to report errors; 50% of respondents reported that their nursing managers demonstrated high levels of coaching. With regard to patient safety culture, areas of strength and weakness were “teamwork within units” (PRR = 66.8%) and “non-punitive response errors” (PRR = 19.7%). Regression analysis findings highlighted a significant association between an intention to report errors and patient safety culture (B = 0.2, CI 95%: 0.1 to 0.3, *P* < 0.05), leader coaching behavior (B = 0.2, CI 95%: 0.1 to 0.3, *P* < 0.01) and nurses’ educational status (B = 0.8, 95% CI: − 0.1 to 1.6, P < 0.05).

**Conclusions:**

Further research is needed to assess how interventions addressing patient safety culture and leader coaching behaviours might increase the intention to report errors.

## Background

According to Sorra and Dyer, patient safety culture (PSC) describes “management and staff values, beliefs, and norms about what is important in a health care organization, how organization members are expected to behave, what attitudes and actions are appropriate and inappropriate, and what processes and procedures are rewarded and punished concerning patient safety” [1]. Safety has been defined as the freedom from accidental injury and error seen in terms of “execution”: the failure of a planned action to be completed as intended or “planning”: the use of the wrong plan to achieve a goal [2]. Such errors can occur at any point in the patient management process, including diagnosis, treatment, and prevention and they may or may not result in an adverse event [3].

Errors risk patients’ health and well-being as well as their lives and can increase the cost of medical treatment, such that the quality of care is negatively affected [4]. James reported that, in US hospitals, a minimum of 210,000 deaths per annum were associated with medical errors [5]. In Australia, each year, 18,000 preventable deaths are attributable to medical errors and at least 50,000 patients are disabled [2], and in Germany 25,000 deaths result from 100,000 medical errors per year [6]. Fundamental to error prevention is the principle that errors should be reported and, to this end, systems have been established to promote error reporting: in Australia and the US in 2000, in the United Kingdom in 2003, and in France in 2006 [3].

In third world and developing countries, accurate estimates are difficult because with no effective recording and reporting systems, there is a shortage of research information. However, it is thought that the medical errors rate is high [7]. In Iran, it is estimated that between 3 and 17% of in-patients experience unwanted side effects as a result of medical errors with 30–70% of these being preventable [8]. Despite such high rates of medical errors, Iranian healthcare organizations have poor levels of reporting [9].

Several factors influence medical error reporting among nurses. One of these is the fear of creating a negative impression by ward staff towards the person who reports an error [10]. Lack of adequate support from colleagues is another factor. Therefore, it is imperative to support health professionals in error-related events [11]. Administrative factors, such as rigidity, cost-cutting measures, lack of policy and standard operating procedure and fault finding were other reasons for under-reporting the errors [12]. Most minor errors and near misses often go unreported [13]. Near misses are often discounted since they pose no harm to the patient. Recognizing and reporting near misses is proactive patient safety and a quality improvement strategy that needs to be adopted in order to prevent similar and harmful events occurring in the future [14].

A review of existing literature found a relationship exists between the number of medical errors reported and elements of PSC [15, 16]. It is evident that leadership is an important element of PSC and that patient safety can be both facilitated and inhibited by perceptions of leadership amongst nurses [17, 18] with a leader’s attitude being reported as a contextual factor in a health care professional’s decision to raise issues in relation to patient safety [19].

Adverse events are seen as providing “information-rich” data for learning and systems improvement by leaders who proactively strengthen PSC [20] and it has been seen that PSC is significantly impacted through education and coaching when leaders follow up on reports that are made [21]. In developing countries, leaders frequently focus their activities on data collection, audit and reporting rather than on catalyzing learning and supporting systems that lead to quality improvement [22, 23]. However, a coaching program has successfully promoted alternative perspectives and supported positive change [24], coaching having emerged as a major tool to continue the education process and enable a change to team-based care [25]. Up-to-date guidance and the support of educators and coaches mean that nurses participate in life-long learning and a culture of safety is created and enhanced [24].

What research there is into leadership coaching for professionals in healthcare settings is anecdotal and a solid evidence-base is yet to be established [26]. However, in Iran, the rate of medical errors in emergency departments is alarming [27]. A recent study in emergency departments has shown that medical errors occurred amongst 46.8% of nurses in emergency departments [28] which are overcrowded, with shortages of staff and equipment, and patients admitted with life-threatening illnesses, all making it more likely that there will be a higher incidence of medical errors [29]. According to a study conducted in the U. S, nearly 3% of all hospital accidents are related to the emergency department [30].

Given this, and the paucity of research exploring the association between PSC, nurses’ intentions to report errors and the coaching behaviour of leaders, [12] this study aims to investigate the relationship between these variables amongst Iranian emergency nurses.

## Method

### Study design

A cross-sectional survey design was adopted for this study.

### Sampling and data collection

A survey was conducted in both public and private hospitals (*N* = 18) in Tabriz, northwest Iran. The study population included 350 nurses, working the morning and evening shifts in 18 emergency departments, with participants identified using census sampling. The inclusion criteria for the sample selection included only staff nurses (a) being a full-time nurse; (b) employment in the emergency department for a minimum of 1 year, and (c) being available during the period of data collection. Those on leave during the study period, nurse educators and nurse managers were excluded from the study.

Institutional consent was obtained prior to data collection after explaining the purpose of the study to the head of the nursing department of each participating hospital. Individual consent was also obtained at the emergency departments from January to March 2019. The purpose of the study was explained to the nurses and the consent forms were filled in by them. The questionnaires were given to those who volunteered to participate in the study. All ethical principles were strictly adhered to, as they were free to withdraw from participating at any point in time, and their participation would not affect their clinical practice. Anonymity was maintained throughout data collection. The completed surveys were retrieved from participants by the designated research assistants in each hospital.

### Study instruments

Tools for gathering data were Demographic and Hospital Survey on Patient Safety Culture (HSOPSC), Coaching Behavior Scale and Intention to Report Errors Questionnaire. All questionnaires were administered in Persian and instruments not already available in this language were adapted to Persian using a standardized back-translation procedure [31] by a panel of experts.

#### Patient safety culture

PSC was measured using the Hospital Survey on Patient Safety Culture (HSOPSC), developed by the Agency for Healthcare Research and Quality [32] to determine nurses’ perceptions of PSC. The HSOPSC questionnaire was translated into Persian in 2012 and has been validated in a previous study [33]. The HSOPSC comprises 12 PSC dimensions, encompassing a total of 42 items, with 3 or 4 items per dimension. All items are measured with a 5-point agreement scale (from 1 = strongly disagree to 5 = strongly agree) or frequency (from 1 = never to 5 = always).

The mean score of each dimension was calculated. Also, a Positive Response Rate (PRR) could be calculated for each item from responses of “strongly agree/agree” or “always/most of the time”. To calculate the PRR of each dimension, the first step was to compute the PRR for each item and then calculate the mean PRR across all items in the dimension. The mean PRRs of the overall HSOPSC can be similarly calculated:

- Scores of 75% and above are considered as representing a good PSC/area of strength.
- Scores between 50 and 75% are considered as a neutral PSC.
- Scores of less than 50% are considered as indicative of a poor/low PSC /need improvement [34].

The HSOPSC was used previously in studies that assessed the perception of staff on the PSC of several Iranian hospitals [33, 35]. In the study conducted by Moghri et al., the Cronbach’s Alpha of the questionnaire was reported to be 0.82 [33] and in this study to be 0.83.

#### Coaching behaviors of the nurse leaders

Leader coaching behaviour (LCB) was measured using the Coaching Behavior Scale, a survey tool designed to assess LCB amongst nurses developed by Stowell [36] and subsequently revised by Ko and Yu [12].

Two independent researchers, with a background in nursing, translated the LCB questionnaire into Persian. The translation was double reviewed and checked by two professors both with background in nursing, leadership and in the English language. The LCB comprises 13 questions scored with a 5-point Likert scale measuring 4 behavioural factors: direction (3 items), development (3 items), performance evaluation (3 items), and relationships (4 items). The total scores range from 13 to 65 points. Higher scores indicate that the coaching behaviour of a manager is perceived as positive.

The validity and reliability of the tool has previously been established by Ko and Yu [12], with good internal reliability at a Cronbach’s alpha coefficient ranging from 0.78 to 0.98. In this study, the Cronbach’s alpha of the LCB was 0.92. The LCB of the nurses was divided into two groups: (high-performance coaching and low-performance coaching). The overall perception of LCB for each respondent was calculated by taking the average scores of the 13 items in the LCB questionnaire. Using this mean score, individuals with a score higher than 3.5 were placed in the high-performance coaching group, and the rest were placed in the low-performance coaching group [12].

#### Intention to report errors

To measure the nurses’ intentions to report their own or others’ errors, we used an instrument developed by Kim [37] which poses three questions: “If you committed an error that had no adverse effect on patients in your current work situation, would you report the error?” “If your colleague committed an error with no adverse effect on patients in your current work situation, would you report the error?” and “Do you share information regarding errors or malpractice with others?” The response options were ‘never’, ‘rarely’, ‘sometimes’, ‘usually’, and ‘always’. In Ko and Yu’s study [12] the Cronbach alpha was 0.83; and in our study, it was 0.76. Previous research has established that appropriate performance of error reporting is indicated by answers that the respondent “always” or “usually” reported their clinical errors and “inappropriate” performance by the responses “sometimes”, “rarely”, and “never” [38].

The demographic variables of the respondents, including age, gender, marital status, education level, work experience (years), and work time (hours per week) were collected at the end of the survey.

### Data analysis

Data analyses were conducted using SPSS 22.0 (IBM Corp., Armonk, NY, USA). The demographic characteristics of the respondents were described using descriptive statistics including frequency, percentage and means and standard deviations. A multiple linear regression model was developed with the intention to report errors as the dependent variable, PSC and LCB as independent variables. The demographic variables were entered to model as potential confounders. The level of significance was set at 0.05.

## Results

Some 279 responses were received over a three-month period. Of these 23 were excluded from the analysis as they were less than 50% complete or did not meet the inclusion criteria. With an overall response rate of 73.1%, a total of 256 questionnaires were analyzed.

Characteristics of the sample are summarized in Table 1. The majority of the sample was female (68.4%) and held a Bachelor’s degree in nursing (54.4%). 54.7% of participants were married. The majority came within the age group 31–40 years (44.5%), and the mean age of the participants was 35.4 (SD = 8.6) years. The average experience in nursing was 10.9 (SD = 7.9) years and 42.2% had been working in nursing for more than 10 years. 53.9% of nurses worked less than 44 h per week and 58.6% were in permanent employment.

**Table 1:** General Characteristics of sample (*N* = 256)

Variables	N (%)
Gender	
Male	81 (31.6)
Female	175 (68.4)
Marital status	
Single	116 (45.3)
Married	140 (54.7)
Age (in years)	
21–30	80 (31.3)
31–40	114 (44.5)
> 40	62 (24.2)
Work experience (in years)	
≤ 5	81 (31.6)
6–10	67 (26.2)
> 10	108 (42.2)
Education level	
Associate degree	13 (5.1)
Bachelor’s degree	147 (54.4)
Master’s degree or PhD	96 (37.5)
Employment status	
Permanent	150 (58.6)
Contract	106 (41.4)
Weekly work time (Hour)	
Normal (≤44)	138 (53.9)
Overtime (> 44)	118 (46.1)

The PRRs and mean (SD) scores of PSC, LCB and intention to report errors are shown in Table 2. Mean (SD) scores for PSC ranged from 2.5 (0.7) to 3.8 (0.7) and the PRRs ranged from 19.7% to 66.8%. The PRRs of PSC dimensions were all less than 75% and the overall PRR was 44.8%. The PRR of “teamwork within units” (PRR = 66.8%) was the highest followed by “manager expectations” (PRR = 65.8%). The PRR of “non-punitive response errors” (PRR = 19.7%) was the lowest. This means that hospital management did not provide a supportive working environment in the promotion of patient safety as workers often preferred not to report errors for the fear of stigmatization, blame and punishment.

**Table 2:** Descriptive statistics of the PSC, LCB and Intention to Report Errors

Variables	Mean (SD)	PRR (%)	Judgment^**a**^
Teamwork within units	3.8 (0.7)	66.8	Neutral
Manager expectations	3.7 (0.9)	65.8	Neutral
Feedback communication about errors	3.7 (0.8)	57.2	Neutral
Staffing	3.4 (0.8)	54.2	Neutral
Events reported	3.3 (0.9)	52.2	Neutral
Management support for patient safety	3.3 (0.9)	48.2	Weakness
Perception of patient safety	3.2 (0.7)	43.8	Weakness
Organizational learning	3.2 (0.7)	42.9	Weakness
Communication openness	3.0 (0.7)	38.1	Weakness
Teamwork across units	2.7 (0.9)	26.6	Weakness
Handoffs and transitions	2.7 (0.6)	22.3	Weakness
Non-punitive response errors	2.5 (0.7)	19.7	Weakness
**Overall PSC**	**2.9 (0.7)**	**44.8**	Weakness
		**High-performance coaching (%)**^**b**^	**Intention to report errors (%)**
Performance evaluation	3.3 (1.0)	55.5	–
Development	3.3 (1.1)	43.8	–
Relationship	3.2 (1.0)	45.7	–
Direction	3.2 (0.9)	35.9	–
**Overall LCB**	**3.3 (0.6)**	**50.0**	–
**Intention to report errors**	**3.4 (0.9)**	**–**	**43.0**

Note: PSC. Patient safety culture, LCB. Leader coaching behavior, PRR. Positive Response Rate

^a^ PRR > 75% was defined as patient safety strength, scores between 50 and 75% are considered as a neutral patient safety and scores of less than 50% are considered as indicative of a poor patient safety

^b^ Score higher than 3.5 were placed in the high-performance coaching group

Mean (SD) scores of LCB ranged from 3.2 (0.9) to 3.3 (1.1). The overall mean (SD) score of LCB was 3.3 (0.6) and of the four dimensions, the highest and lowest perceived coaching performance related to “performance evaluation” (55.5%) and “direction” (35.9%). The mean (SD) score of intention to report errors among nurses in this study was found to be 3.4 (0.9). Of the total participants (*n* = 256), 43% reported that they had a high intention to report errors.

Table 3 shows the results of multiple linear regression analysis which was used to predict nurses’ intention to report error.

**Table 3:** Multiple linear regression analysis of factors associated with intention to report error (N = 256)

Variables	Beta (95% CI)
Patient safety culture^*^	0.2 (0.1 to 0.3)
Leader coaching behavior^**^	0.2 (0.1 to 0.3)
Age (reference: > 40)	
21–30	0.1 (− 0.3 to 0.5)
31–40	0.2 (− 0.1 to 0.5)
Gender (reference: female)	− 0.1 (− 0.3 to 0.2)
Marital status (reference: married)	0.1 (− 0.1 to 0.3)
Education level (reference: Masters or PhD degree)	
Associate degree^*^	0.8 (− 0.1 to 1.6)
Bachelor	0.6 (− 0.1 to 1.3)
Employment status (reference: Contract)	− 0.2 (− 0.5 to 0.1)
Work experience (reference: > 10)	
≤5	−0.2 (− 0.5 to 0.1)
6–10	−0.3 (− 0.6 to 0.1)
Work hours (reference: overtime)	0.1 (− 0.1 to 0.3)
R^2^ = 4.7% F = 15.3 *P* < 0.001	

Dependent Variable: intention to report error

* indicates significant value (p < 0.05)

** indicates significant value (p < 0.01)

A statistically significant difference was shown between the educational level of nurses and their intention to report errors. Nurses with associate degree education were 80% times more likely to report errors than those with Bachelor, Masters or PhD degree (B = 0.8, 95% CI: − 0.1 to 1.6, *P* < 0.05). No significant relationship was found in relation to other demographic characteristics. An increase of 20% in the intention to report errors was observed for a one unit increase in the score on PSC (B = 0.2, CI 95%: 0.1 to 0.3, P < 0.05). Similarly, an increase of one unit in the score on LCB, the intention to report error was increased by 20% (B = 0.2, CI 95%: 0.1 to 0.3, *P* < 0.01).

## Discussion

This study examined the relationship between emergency nurses’ perception of PSC and LCB with their intention to report errors. The results show that, based on PRR scores, none of the 12 dimensions achieved scores of 75% and cannot, therefore, be considered to represent areas of patient safety strength. This result is in contrast to findings of other research [39]. It was also lower than other studies conducted in countries including Taiwan [40], Lebanon [16] and Saudi Arabia [41], with cultural and organizational differences relating to patient safety thought to explain the differences.

Perhaps one of the most important factors to mention in the same studies is the disparity in accreditation policies and procedures in three countries where the study was conducted. For instance, there is a mandatory accreditation system in the Iranian health system monitored by the Ministry of Health which has not fully taken shape, while Lebanon and Saudi Arabia were among the countries in the Eastern Mediterranean region whose accreditation standards have been approved by the International Society for Quality in Health Care (ISQUA) and are monitored by international organizations [42].

Another challenge of the Iranian healthcare system is staff shortages, the financial pressures experienced by hospitals, lack of senior management support for patient safety culture and lack of systematic approach for reporting errors [43, 44] which means patient safety is seen as a low priority by managers. For patient safety to be effective, there is a need for continuous educational advancement at every level of the organization. In addition, provision of necessary infrastructure, resources (human, financial, technological and material) and procedures necessary for the development of patient safety culture needs to be implemented [45].

A previous Iranian study conducted in an academic intensive care unit [46], like the results in this study, found that all dimensions needed to be improved. These findings contrast with those of Habibi et al. (2016) where a higher PRR score was found in teaching hospitals in Tehran [47]. A recent Iranian systematic review illustrates that, compared to the results of studies conducted in other countries, the mean of the responses in Iran for the different dimensions of PSC is low, a finding which underlines the fact that, for many people working in Iranian hospitals (including the managers), the concepts of PSC are unknown [48]. This is possibly because, rather than the issue being neglected, PSC is a relatively new concept in Iranian hospitals and has not been fully recognized [49].

The dimension with the highest PRR was “teamwork within units”. Whilst this reflects the findings of other studies [10, 50], in our study it was an area of patient safety weakness. “Non-punitive response to error” had the lowest PRR, a finding which follows an earlier study conducted in a public hospital in Tabriz and which examined the same issues [51]. These findings are consistent with other local findings [47] and those from international studies [10, 16, 52], and would suggest that a major barrier to error reporting is the risk of a punitive response. When non-punitive measures are taken, errors will be detected and reported early and further occurrences will be prevented [53].

Punishing staff for their mistakes has been a strong measure taken by administrators and senior colleagues in many Iranian hospitals, without considering the reasons for such errors. This policy has affected continuous education and the work environment at large [48]. For example, nurses in this study, like those in other similar studies, felt that if they reported their errors, a record of their mistakes would be held in their personal file and may be used against them at some point in the future and, for this reason they preferred silence over-reporting errors.

It is of interest that 50% of nurses in this study tended to rate their managers’ coaching behaviour as high. In line with the study conducted by Ko and Yu [12] the highest and lowest perceived LCB in this study was attached to “performance evaluation” and “direction”. It is important to note that, in respect of “performance evaluation”, only half of the participants described their leaders as being high-performing coaches and that in respect of “direction” the percentage was 35.9%. Given the evidence that a lack of performance appraisal can impact negatively on nurse performance [54] and that coaching on the part of team leaders supports learning from problems and errors amongst members [55], it can be concluded that the perceived coaching behaviour in this study may impact negatively on nurse performance in respect of safety-related issues.

This study found that, overall, 43% of nurses had a high intention to report errors, a similar finding to those of earlier studies in other countries [56–58] in which it was demonstrated that the proportion of error reporting amongst nurses was less than 50%. These findings are significant as there is evidence which suggests that whilst nurses intercept 86% of potential errors [59],between 34 and 50% don’t report medical incidents [60].

In looking to explain the low rates found in these studies, it is possible that an intention to report is linked to an attitude towards reporting and an awareness of reporting, as well as the existence of support [4]. There are also a multitude of reasons, including fear, humiliation, a punitive reporting culture and limited follow up, following error reporting, that may lead to under-reporting [10]. Having said this it was found, in an Ethiopian study, that the proportion of error reporting amongst nurses was 57.4% [61], a difference that may be related to differences in error reporting systems and to differences in the time frame in which the studies were conducted.

Human behaviour is influenced by motivators which are borne out of their intentions, which show peoples’ willingness and commitment to their actions and behaviour [62]. Ajzen (1991) explained this in the Theory of Planned Behavior (TPB) that intention can predict an individual’s needs and it has been confirmed in many studies [63]. According to the TPB, intention mediates between attitude and actual behaviour or performance [62].

This study found a significant association between nurses’ intention to report errors and the level of their education. Those nurses with an associate degree education were 78% more likely to report errors than nurses at a different educational level. This may be because professional nurses have a fear of legal consequences or of losing their occupational position [10]. In contrast, a study conducted by Poorolajal et al. (2015) found that managers and staff who had attained higher educational levels had a greater willingness to report errors [9]. Another study also revealed that reporting medical errors depends on individual’s marital status [64]., while this is not confirmed in our study.

Nurses who experienced a high level of PSC were found to be more likely to report errors in this study, a finding which reflects that of Kagan et al. (2013) whose Israeli study confirmed that a readiness to report errors was influenced by an organization’s safety culture [58]. Furthermore, a flexible culture can promote patient safety and error reporting within an organization by developing trust and improving the problem-solving capabilities of nurses [12].

This study also found that nurses who saw their managers’ coaching as being at a high level of performance reported a stronger intention to report errors, a finding which follows that of Ko and Yu [12]. In nursing, a manager develops capabilities by exposing nurses to appropriate coaching strategies which together with regular feedback encourages them to work independently [65]. As has been pointed out by Reid Ponte et al. [66] nurses who have experienced coaching describe it as helping them to recognize and modify behaviours that have hampered their performance, and in doing so, improve their effectiveness and that of the organization.

### Strengths and limitations

This study has several strengths. Notably, it is the first study to have investigated the LCB of Iranian nurses, using validated tools to measure variables with a homogeneous study population. However, the study also has limitations. Participants in the study were emergency nurses working in hospitals in Tabriz, Iran and, as such, the results may not be generalized to other hospitals or different clinical settings. Given such limitations, further studies in settings other than an emergency department may be required if the findings of this study are to be fully justified. Furthermore, as nurses were the focus of this study a more complete picture might be obtained if other studies focusing on other staff were conducted.

Besides, as this study adopted a cross-sectional approach and did not seek to establish cause and effect, it is recommended that further studies adopt a longitudinal evaluation. It is also the case that potential organizational factors and a blame culture that were both identified in this study would benefit from a further in-depth study in which a qualitative approach was adopted. Finally, the near misses are counted as insignificant since there is no harm to the patient and because of poor research evidence, due to ineffective recording and reporting systems in developing countries such as Iran, this study has measured the intention to report errors, instead of the actual number of errors reported. Therefore, it is suggested to measure numbers of errors reported in future studies.

## Conclusion

In this study the intention to report errors among nurse respondents was low. Given that a high perception of PSC and LCB increases nurse intention to report error, it seems that hospital managers and nursing administrators have an important role to play. They have the power to shape the working environment, in terms of removing barriers to error reporting and providing a supportive environment so that nurses feel they can report errors without fearing reprisals. Given that the greatest contributor to low levels of PSC relates to “non-punitive response errors” and the fact that a fear of punishment has consistently been found to reduce the frequency of error reporting, it is incumbent on health decision-makers to adopt programs that create an atmosphere in which individuals can openly discuss medical errors and potential hazards.

Further, a culture which sees errors as an opportunity to improve a system should replace a blame culture, in which errors are seen as personal failures. Indeed the usefulness of education and of efforts towards developing a culture which encourages the reporting of patient safety issues is evident. Neither should it be forgotten that nurses who perceived the manager’s coaching as being of a high level of performance reported a stronger intention to report errors. Medical errors cause patients across the world to suffer disabling injuries and leadership coaching could be a significant means by which error reporting is facilitated, thereby benefiting not only patients and their families, and those that work in the health service, but also the wider community.

## Data Availability

The datasets analyzed during the current study are available from the corresponding author on reasonable request.

## Abbreviations

PSC: Patient safety culture
HSOPSC: Hospital Survey on Patient Safety Culture
PRR: Positive Response Rate
LCB: Leader coaching behaviour
SPSS: Statistical Package for Social Sciences
TPB: Theory of Planned Behavior
